# Rai1 Haploinsufficiency Is Associated with Social Abnormalities in Mice

**DOI:** 10.3390/biology6020025

**Published:** 2017-04-27

**Authors:** Nalini R. Rao, Clemer Abad, Irene C. Perez, Anand K. Srivastava, Juan I. Young, Katherina Walz

**Affiliations:** 1 John P. Hussman Institute for Human Genomics, University of Miami, Miami, FL 33136, USA; nalini.rao326@gmail.com (N.R.R.); cabad@med.miami.edu (C.A.); irene.perez28@gmail.com (I.C.P.); jyoung3@med.miami.edu (J.I.Y.); 2 J.C. Self Research Institute, Greenwood Genetic Center, Greenwood, SC 29646, USA; anand@ggc.org; 3 Dr. John T. Macdonald Foundation Department of Human Genetics, Miller School of Medicine, University of Miami, Miami, FL 33136, USA

**Keywords:** Autism spectrum disorder, Smith-Magenis syndrome, *Rai1*, social behavior

## Abstract

*Background:* Autism is characterized by difficulties in social interaction, communication, and repetitive behaviors; with different degrees of severity in each of the core areas. Haploinsufficiency and point mutations of *RAI1* are associated with Smith-Magenis syndrome (SMS), a genetic condition that scores within the autism spectrum range for social responsiveness and communication, and is characterized by neurobehavioral abnormalities, intellectual disability, developmental delay, sleep disturbance, and self-injurious behaviors. *Methods:* To investigate the relationship between *Rai1* and social impairment, we evaluated the *Rai1^+/−^* mice with a battery of tests to address social behavior in mice. *Results:* We found that the mutant mice showed diminished interest in social odors, abnormal submissive tendencies, and increased repetitive behaviors when compared to wild type littermates. *Conclusions:* These findings suggest that *Rai1* contributes to social behavior in mice, and prompt it as a candidate gene for the social behaviors observed in Smith-Magenis Syndrome patients.

## 1. Introduction

The difficulties and deficits observed in autism span a wide range as the disorder is characterized by various phenotypes. The spectrum of these phenotypes includes: difficulties in social interaction, verbal and nonverbal communication, and repetitive behaviors. Autism is recognized as an entity by itself, but autistic features are also present in several neurodevelopmental syndromes such as: Rett, Fragile-X, Angelman, Prader-Willi, Joubert, Tuberous sclerosis, and 22q13.3 deletion syndromes, among others [1]. One of those syndromes that includes autistic features is Smith-Magenis syndrome (SMS; Mendelian Inheritance in Man number (MIM), 182290).

SMS is a microdeletion syndrome with an estimated incidence of 1:15,000 to 1:25,000 live births [2,3]. The phenotypes more commonly associated with SMS are a variable degree of intellectual disability, speech and motor delay, behavior abnormalities with sleep disturbances, self-injurious and/or aggressive attention-seeking behavior, and distinctive craniofacial and skeletal anomalies [3]. SMS is typically caused by a deletion of chromosome 17p11.2 that encompasses multiple genes, including the retinoic acid-induced 1 (*RAI1*) gene or heterozygous mutation in the *RAI1* gene [4,5,6,7,8,9,10,11].

SMS patients harboring the common full deletion have also been described to have stereotypies, sensory integration difficulties, and social communication problems consistent with select features of autism spectrum disorder (ASD) [12,13,14,15,16]. In a recent study, 90% of 26 individuals with SMS showed social responsiveness scale (SRS) scores consistent with ASD [13]. Moreover, the social responsiveness questionnaire scores consistently showed that a majority of individuals may meet the criteria of ASD at some point in their lifetime [13]. However, the likely contribution of the *RAI1* gene to autistic features remains unknown.

Mice are very useful for understanding genotype-phenotype correlations. Studies in chromosomally engineered mice that carried a deletion of the SMS syntenic region in chromosome 11 (*Df (11)17/+*) showed that these animals replicated most of the features observed in SMS patients, including abnormal social behaviors [17,18,19]. Mice carrying an inactivated *Rai1* allele (*Rai1^+/−^*) also recapitulate several SMS features but their social behavior was not yet investigated [19,20,21,22].

In order to understand the contribution of *RAI1* to social behavior, we tested mice carrying an inactivated *Rai1* allele (*Rai1^+/−^*). The *Rai1^+/−^* mouse model showed diminished interest in social odors, abnormal submissive tendencies, and increased repetitive behaviors when compared to wild type littermates, strongly suggesting a role of *RAI1* in social behaviors.

## 2. Materials and Methods

### 2.1. Animals

Heterozygous mice carrying an inactivated *Rai1* allele were kindly donated by Dr. J.R. Lupski and backcrossed more than 12 times to wild type C57BL/6-*Tyr^c-Brd^*. Mice were genotyped as previously described [22], and at the time of weaning, were housed in a cage with two to five animals of mixed genotypes. Housing conditions included a 12 h light: dark cycle (lights on at 6 AM, off at 6 PM) with access to food and water ad libitum. All animal testing was performed in accordance with the guidelines for the care and use of laboratory animals of the United States Public Health Service, National Institutes of Health, and was approved by The Institutional Animal Care and Use Committee at the University of Miami. The National Institutes of Health (NIH) Office of Laboratory Animal Welfare Assurance number is A3224-01. Behavioral testing of male mice began at 8 weeks of age, and all testing was performed between 9 AM–1 PM. The number of mice analyzed for all tests was *Rai1^+/−^* = 13 and wild type = 12 unless otherwise specified. Prior to each test, a habituation period of 30 min was given to the testing environment. The testing battery lasted five days and each day a single test was performed in the following order.

### 2.2. General Health and Neurological Behavior

Mice were evaluated for coat condition and whiskers. The visual placing reflex was carried out by elevating the mouse and then placing it on a bench. If the mouse placed both feet on the bench, the reflex was considered normal. Vibrissae orienting was performed as previously reported [23]. Throughout this evaluation, the vocalization of the mouse was recorded.

### 2.3. Self-Grooming in A Novel Environment

Each mouse was placed in a new cage without bedding for a habituation period of 10 min. Following habituation, an observer with a stopwatch recorded the amount of time the mouse spent self-grooming out of a total time of 10 min.

### 2.4. Sociability and Social Preference

We tested for sociability and social novelty using a rectangular three-chamber apparatus composed of clear carbonate [24]. This equipment contained free access to every chamber. The test consisted of three 10 min sessions, and was performed as previously described [23]. Briefly, in the first 10 min session, the mice were allowed to freely explore the three-chamber equipment. In the second 10 min session, an empty container was placed in the center of one chamber while an unfamiliar stranger mouse (stranger 1) was placed under a container on the other side of the chamber. The test mouse was located in the center chamber and allowed free access to the three chambers. The stranger 1 and the empty container were alternatively changed between chamber sides. Finally, in the third 10 min session, a new unfamiliar stranger mouse (stranger 2) was placed under the formerly empty container. The test mouse was placed in the center chamber with access to each chamber. For each of the three 10 min sessions, the total time spent in each was recorded. To exclude any environmental interference during the testing, the stranger 1 and the empty cage (during the sociability part of the test, or the stranger 1 and stranger 2, during the preference for social novelty test) were placed alternatively in the left or right side of the test chamber, and no significant difference in time spent within any of the chambers for any genotype was observed [25] (*p* > 0.05).

### 2.5. Interest in Non-Social and Social Odors

Each mouse was placed in a new cage with bedding for a habituation period of 60 min in the testing environment. Following habituation, each mouse was presented first with a cotton swab embedded with water for training. Then two non-social odors and two social odors were presented to them three times at separate intervals of 2 min, with 1 min in between. The non-social odors were almond and banana extract diluted 1:100 in water. The novel social odors were obtained from two male mice of the same strain that were housed in a separate cage for more than two days. A cotton swab was immersed in each non-social odor and then placed 4–5 cm from the bottom of the cage. For the social odor, a cotton swab was swiped in a zigzag pattern across the bottom of each dirty cage that housed the unfamiliar mouse. The experiment was video-recorded and the amount of time the mouse sniffed within 3 cm of the cotton swab was documented.

### 2.6. Dominance Tube Test

Mice were trained to enter a clear acrylic tube from both sides and exit towards their respective opposite sides. The training time for each mouse was recorded. After training, two mice, both unfamiliar with each other, were allowed to enter the tube at the same time at opposite sides. An observer recorded the time it took for one mouse to cause the other to back out of the tube. The mouse that did not back out was classified as the winner. This was repeated again in round 2, changing the side of entry into the tube for each mouse [23].

### 2.7. Statistical Tests

Statistical analysis of weight, self-grooming time, and smelling time was performed utilizing the two-tailed Student’s t-test and a *p*-value ≤ 0.05 was considered significant. The dominance test was analyzed with the *X*^2^ statistical test. Two-way (genotype X side) ANOVA (Analysis of variance) with repeated measure (side) followed by a *t*-test analysis when a significant *F*-value was determined was utilized to analyze the sociability and social novelty preference data.

## 3. Results

### 3.1. Abnormal Social Behaviors in Rai1^+/−^ Mice

The clinical presentation between SMS patients harboring a common genomic deletion of ~3 Mb and patients with a point mutation in *RAI1* is very alike. Despite the high incidence of autism in the group of patients harboring the common deletion (~90%) the incidence of autism in the group of patients carrying a *Rai1* point mutation is still unknown (Table 1). On the other hand, *Df(11)17^+/−^* and *Rai1^+/−^* mice were extensively studied and they both recapitulate SMS features very similarly (Table 2), making the *Rai1^+/−^* mouse a good model to study the contribution of *RAI1* to the SMS phenotype. To understand the relationship between *Rai1* and social behaviors, we examine the social responses of *Rai1^+/−^* mice. We utilized a set of specific tests that have been validated in mouse models and are relevant to human autistic behaviors [26]. We first tested our mice for general health and neurological reflexes, such as coat condition, piloerection, presence of full whiskers, visual placing reflex, and the vocalization during handling. No significant differences were found between the *Rai1^+/-^* mice and their wild type littermates for any of these parameters [25].

A comparison of phenotypes patients with the common 17p11.2 deletion and those with intragenic mutations within *RAI1* is shown. The percentages of occurrence for each of the phenotypes are given. NR = phenotype not reported.

We tested the sociability of *Rai1^+/−^* and wild type mice as well as their preference for social novelty, utilizing the three-chamber test, which is based on measuring the place preference (in time spent) for the mice that reflects the innate propensity of a mouse to spend more time in proximity to another animal instead of an inanimate object [23,24]. The percentage of time spent for each genotype in each compartment during the habituation period was analyzed to check for any environmental interference within the social test chamber. No chamber preference was apparent for any genotype (*p* > 0.05).

**Figure 1:**
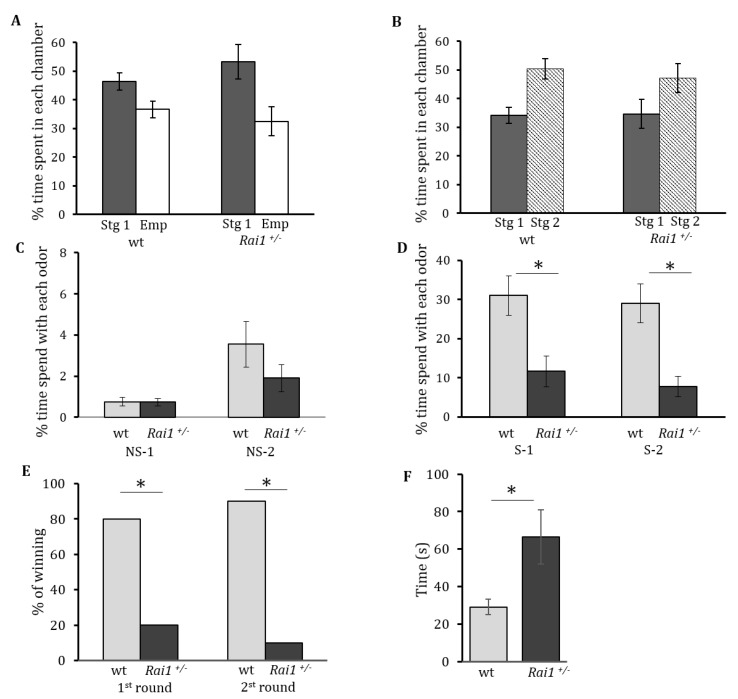
Evaluation of social behaviors in *Rai1^+/-^* mice. (**A**) Sociability in *Rai1^+/−^* mice is shown. Percentages of time spent in the chamber side with stranger 1 (Stg1, black column) or with the empty container (Emp, white column) during the sociability test are shown for the wild type and *Rai1^+/−^* mice. (**B**) Social novelty preference was evaluated. Percentages of time spent in the chamber side with stranger 1 (Stg1, black column) or with stranger 2 (Stg 2, striped column) during the preference for social novelty test are depicted. (**C**) The amount of time spent smelling non-social (NS) odors for both genotypes is shown. NS1 = Almond, NS2 = banana. (**D**) The amount of time spent smelling social (S) odors for both genotypes is shown. (**E**) Tube test. The results for the first and second round are depicted as the percentage of winning for each genotype. Wild type mice (grey column) and *Rai1^+/−^* mice (black column) are represented. (**F**) Total time spent self-grooming for each genotype is shown. Wild type animals are represented in the grey column and *Rai1^+/−^* mice in the black column. Asterisk denotes significant difference (* *p* < 0.05). The mean ± SEM values are depicted.

The analysis of the sociability data showed no significant interaction between the genotype X side (*F*_(1, 59)_ = 1.94, *p* = 0.17). No significant effect on genotype was found (*F*_(1, 59)_ = 13.1, *p* = 0.0006), but a main effect of chamber side (*F*_(1, 59)_ = 0.12, *p* = 0.73) is present (Figure 1A) indicating that both genotypes are equally sociable.

The second part of the test was done to evaluate the preference for social novelty of the different genotypes. We analyzed the preference for social novelty data and observed no significant effect of genotype (*F*_(1, 59)_ = 0.1, *p* = 0.75) nor interaction genotype X side (*F*_(1, 59)_ = 0.96, *p* = 0.33). A significant main effect for side (*F*_(1, 28)_ = 8.77, *p* = 0.0045), was present, suggesting no difference on the preference for social novelty between mutant and wildtype mice (Figure 1B).

Olfactory clues are essential to mouse communication. In addition, a measure of social interest can be obtained by examining the amount of time spent by a mouse smelling non-social and social odors. Here we compared the interest in non-social or social odors between both genotypes by presenting mice with two non-social odors: almond (NS-1) and banana (NS-2), and two different social odors (S-1, S-2). The time spent from the first two trials of each respective odor was averaged and a two-tailed Student’s t-test was performed to analyze the data. There was no significant difference in the time spent interested in non-social odors between both genotypes, for NS-1 (*p* = 0.96) and for NS-2 (*p* = 0.21) (Figure 1C). However, when the mice were exposed to social odors we found that *Rai1^+/−^* mice spent significantly less time smelling the social odors compared to their wild type littermates (S-1 *p* < 0.001, S-2 *p* < 0.001). This reinforces the idea of an abnormal social response for the mutant mice (Figure 1D).

To further evaluate social interactions we used the tube test, a paradigm previously found to be useful in predicting social behavior abnormalities [23,28]. When confronted by another mouse coming from the opposite end of a tube in which they cannot turn around, wild type mice explore each other for a short while and then randomly push the opposing mice, or retreat the tube by walking backwards. However, as can be seen in Figure 1E, when *Rai1^+/−^* mice confronted wild type mice, they backed out of the tube more times than expected by chance (80% of the time) compared with the wild type mice (20% of the time). This test was repeated with the same pair of mice for a second round, switching the side from which each mouse starts. As can be seen in Figure 1E, *Rai1^+/−^* mice again backed out significantly more than expected (90% of the time), compared with the wild type mice (10% of the time) eliminating the possibility of tube side bias. As each *Rai1^+/−^* mouse required a wild type opponent, n = 12 was used for *Rai1^+/−^* and wild type groups. These results of the chi-squared test performed showed that they were significantly different (*X*^2^, *p* < 0.001).

No aggressive behaviors during the encounters were observed. No differences were found between *Rai1^+/−^* and wild type mice in the time they took to walk out of the tube during training. In addition to this, no difference was found in strength or in weight between the groups, suggesting increased anxiety in a novel arena when faced with a non-familiar mouse.

### 3.2. Repetitive Behaviors Were Augmented in Rai1^+/−^ Mice

Repetitive behaviors in this study were evaluated by measuring the time spent self-grooming in a novel environment during a 10 min period. A significant increase in self-grooming time was found for *Rai1^+/−^* (66.6 +/− 14 s) animals compared to their wild type littermates (29 +/− 4.5 s) (*p* < 0.03) (Figure 1F), suggesting an increase of repetitive behaviors in the mutant mice.

## 4. Discussion

SMS is a neurodevelopmental disorder with a clinical presentation that includes craniofacial dysmorphic features, abnormal circadian rhythm, and cognitive impairment with behavioral and psychiatric symptoms. In addition, individuals with SMS have also been described to have stereotypies, sensory integration difficulties, and social communication problems consistent with select features of ASD [13]. Several heterozygous *RAI1* gene mutations have been found to be associated with SMS. These mutations include nonsense, missense, and frameshift mutations caused by deletions of one or multiple nucleotides [5,6,7,8,9,10,11]. In addition, the *RAI1* gene has also been associated with spinocerebellar ataxia (SCA2), schizophrenia, and autism [29,30,31].

We have previously developed and described a mouse model for SMS, *Df(11)17^+/-^* mice, carrying a genomic deletion of ~23 genes in the mouse genomic region syntenic to the human deleted region [17]. This mouse model recapitulates several physical and behavioral aspects present in SMS, including social behavior abnormalities [18,19]. Here we explore the role of *Rai1* in the social phenotype of the mice. We show that haploinsufficiency of the *Rai1* gene is enough to cause some abnormal social behaviors in mice (Table 2) utilizing very standardized tests that were previously reported to study autistic like behaviors in mice [26].

Social interaction in mice is heavily based on the reliance of olfactory cues. In the olfactory test there was no significance difference between wild type and *Rai1^+/−^* mice in the time spent smelling non-social cues, indicating that olfactory function in mutant mice is normal. However, *Rai1^+/−^* mice showed a lack of interest in social odors when compared to wild type littermates suggesting an impaired discrimination of social olfactory cues. This same phenotype was observed in mutants for *Tsc1* [32], a mouse model for Tuberous Sclerosis syndrome, a genetic disorder with high rates of comorbid ASDs.

The grooming test is utilized to investigate repetitive behaviors in mice. *Rai1^+/−^* mice showed increase repetitive behaviors in the grooming test when compared with their wild type littermates, suggesting an increase of stereotypic behaviors. Significantly more self-grooming time was also found in other mouse models as mutants for *Shank2* [33], *Tsc1* [32], *Neurexin 1α* [34], *En2* [35], and *Ankrd11* [36], all candidates for autistic like behaviors.

The tube test is used as a measure for social dominance. The results showed that the *Rai1^+/−^* mice displayed abnormal submissive tendencies, in concordance to the phenotype previously seen for *Df(11)17^+/−^* mice in this same test [19]. Again this phenotype was observed in different mouse models for autistic candidate genes as mutants for *Fmr1* [37].

To test for sociability and preference to social novelty we utilized the three-chamber test. No significant difference was found between wild type and *Rai1* mutant mice. Recently, a new mouse model of *Rai1* deficiency was developed where *Rai1* ablation was done conditionally in different neuronal populations [38]. In this mouse model the three chamber test result was similarly not significant. Interestingly enough, some other mouse models of autism were negative in the three chamber test as *Nlgn3* knock-in mouse [39] and *Fmr1* [40]. The behaviors measured in the three-chamber test may not be correlated to reciprocal social interaction in a naturalistic environment, therefore these two groups of mice may behave differently in a home cage behavior analysis [37,41,42]. Also, it may not be clear if the approach toward a caged mouse reflects an affiliative social approach or aggressive behavior, but during observation of our three-chamber test, it is more likely that the approach toward the stranger mouse reflects affiliative social behavior as the subjects did not demonstrate any aggressive behavior phenotypes such as: tail rattling, chasing, or aggressive attacks [43]. It should also be noted that mice by nature are territorial and the design of this experiment does not allow for direct interaction in this apparatus therefore we are unable to state if this behavior is reflective of ASD-related behavior.

A more suitable behavioral test to investigate social interactions in addition to the three-chamber test would be a home-cage social interaction test and a novel environment social interaction test [44]. While we are unable to state if this test is reflective of ASD-related behavior, the results of the three-chamber test suggest that the wild type and *Rai1* mutant animals behave in the same manner in this behavior test.

We have shown here that *Rai1* haploinsufficiency is enough to cause several social abnormalities in mice. These results parallel those previously seen with various other mouse models for social behavior abnormalities, as summarized in Silverman et al, 2010 [26]. Most *Rai1^−/−^* mice died during embryonic development [22], however, it was possible to show that *Rai1^−/−^* mice that survive present a more severe phenotype than *Rai1^+/−^* mice, including the learning disability, motor deficiency, and seizures [21]. It would be interesting to see the effect of *Rai1* knock out on social behavior.

In summary, our data shows that *Rai1* is responsible for social abnormalities in mice, and reinforces the idea that *Rai1* should be considered a candidate gene in children with autistic manifestations or social abnormalities.

## 5. Conclusions

Our data shows that Rai1 is responsible for social abnormalities in mice, and reinforces the idea that *Rai1* should be considered as a candidate gene in children with autistic manifestations or social abnormalities.

## Figures and Tables

**Table 1:** Comparison of the clinical presentation of SMS patients with common deletion and *RAI1* mutations.

	SMS Patients	
Phenotypes	% in Common 17p11.2 Deletion [3,8,11]	% in *RAI1* Mutations [5,8,9,11]
Craniofacial Abnormalities	100	100
Skeletal Abnormalities		
Short stature	70–80	11
Scoliosis/ Vertebral Abnormalities	73	40–50
Short broad hands/Brachydactyly	85	88
Otorhinolaryngological		
Hoarse Voice	66–80	76–86
Hearing loss	60–68	11–33
Neurological		
Cognitive Impairment	100	100
Infantile hypotonia	<90	50–61
Speech delay	>90	70
Motor delay	>90	60-70
Sleep disturbance	90	100
EEG abnormalities	50–66	80
Seizures	11–30	16.6–50
Behavioral		
Self-hugging	50–80	100
Onychotillomania	25–85	80–100
Polyembolokoilamania	25–85	75–80
Head banging/face slapping	70	90
Hand biting	80	60–71
Attention seeking	80–100	100
Aggressive behavior		55
Self-injurious behavior	70–90	>90
Hyperactivity	80	100
Autistic features	90 [7]	NR
Other features		
Cardiac defects	30	0
Renal/urinary tract defect	30	0
Obesity	18	78
Overeating	25	81

EEG = Electroencephalogram; NR = phenotype not reported.

**Table 2:** Comparison of neurological phenotypes in mice with the syntenic common 17p11.2 deletion and those with a *Rai1* mutation.

	SMS Mouse Models	
Phenotypes	Common Deletion [17,18,27]	*Rai1^+/−^* [19,22]
Neurological		
Overt seizures	Yes (~20%)	Subtle
EEG	Abnormal	Abnormal
Locomotor activity ^+^	Decreased	Decreased
Anxiety ^+^	Normal	Normal
Learning and memory	Normal	Normal
Social novelty recognition ^&^	Abnormal	Normal *
Social (dominant behavior) ^#^	Decreased	Decreased *
Repetitive behaviors ^++^	NR	Increased *

The * denotes phenotypes described in this paper. + = Open field test. & = Three-chamber social interaction test. # = Tube test. ++ = Grooming Test. NR = phenotype not reported.
